# Core-shell Au-Pd nanoparticles as cathode catalysts for microbial fuel cell applications

**DOI:** 10.1038/srep35252

**Published:** 2016-10-13

**Authors:** Gaixiu Yang, Dong Chen, Pengmei Lv, Xiaoying Kong, Yongming Sun, Zhongming Wang, Zhenhong Yuan, Hui Liu, Jun Yang

**Affiliations:** 1 Key Laboratory of Renewable Energy, Chinese Academy of Sciences, Guangzhou Institute of Energy Conversion, Chinese Academy of Sciences, Guangzhou 510640, China; 2 Guangdong Key Laboratory of New and Renewable Energy Research and Development, Guangzhou Institute of Energy Conversion, Chinese Academy of Sciences, Guangzhou 510640, China; 3 State Key Laboratory of Multiphase Complex Systems, Institute of Process Engineering, Chinese Academy of Sciences, Beijing 100190, China

## Abstract

Bimetallic nanoparticles with core-shell structures usually display enhanced catalytic properties due to the lattice strain created between the core and shell regions. In this study, we demonstrate the application of bimetallic Au-Pd nanoparticles with an Au core and a thin Pd shell as cathode catalysts in microbial fuel cells, which represent a promising technology for wastewater treatment, while directly generating electrical energy. In specific, in comparison with the hollow structured Pt nanoparticles, a benchmark for the electrocatalysis, the bimetallic core-shell Au-Pd nanoparticles are found to have superior activity and stability for oxygen reduction reaction in a neutral condition due to the strong electronic interaction and lattice strain effect between the Au core and the Pd shell domains. The maximum power density generated in a membraneless single-chamber microbial fuel cell running on wastewater with core-shell Au-Pd as cathode catalysts is ca. 16.0 W m^−3^ and remains stable over 150 days, clearly illustrating the potential of core-shell nanostructures in the applications of microbial fuel cells.

Microbial fuel cells (MFCs), which combine the developments in biotechnology with fuel cell sectors, have garnered sustained research interest due to their immense potential to pollutant removal, while directly produce electricity for various small electronic devices, e.g. sensors, pumps, clocks, and mobile phones^1^,^2^,^3^,^4^,^5^,^6^,^7^,^8^,^9^,^10^. In a typical air-cathode MFC, the bacteria are immobilized on the anode to oxidize organic compounds from wastewater stream to carbon dioxide (CO_2_), while oxygen reduction reaction (ORR) occurs at the cathode to combine with protons transferred through the electrolyte. Suffering from the low stability (as from poisoning by contaminants) and high costs (e.g. platinum-based materials), the materials used to catalyze ORR at cathode has become one of the barriers to the commercialization of MFCs^11^,^12^,^13^,^14^,^15^. In addition, different from the chemical fuel cells, in which strong acid or alkaline is often used as electrolyte, in a MFC, the microbial proliferation usually requires the electrodes to be immersed in a solution with neutral pH value, which leads to kinetic sluggishness of ORR and large overpotential at cathode^16^. Hence, although a large number of successes have been achieved in normal chemical fuel cells, the widely used Pt-based materials might not be suitable as cathode catalysts for MFC systems^17^. Up to date, extensive research efforts have been devoted toward the evaluation of carbonaceous materials and inexpensive transition metals as ORR catalysts in MFCs^18^,^19^,^20^,^21^,^22^,^23^. However, besides their cost advantage, these alternatives have unacceptable activity and durability for oxygen reduction compared with Pt-based materials. In this sense, searching high efficient ORR catalysts is still necessary for the practical use of MFCs.

This work aims at the exploration of relatively inexpensive Pd-based nanomaterials as cathode catalysts for MFCs. We report the synthesis of bimetallic Au-Pd nanoparticles with a core-shell construction and investigate their catalytic properties toward ORR. We will demonstrate that, under neutral conditions, the bimetallic core-shell Au-Pd nanoparticles exhibit much better activity and durability for ORR than those of hollow Pt nanostructures. The maximum power density generated in a membraneless single-chamber MFC running on wastewater with core-shell Au-Pd as cathode catalysts is 15.98 W m^−3^ and remains stable over 150 days, clearly illustrating the potential of core-shell nanostructures for the applications of MFCs. The complex electronic interaction and the lattice strain generated between the Au core and Pd shell may tune the d-band center of Pd atoms, and accounts for the observed ORR enhancement of core-shell Au-Pd nanoparticles.

## Result and Discussion

**Figure 1:**
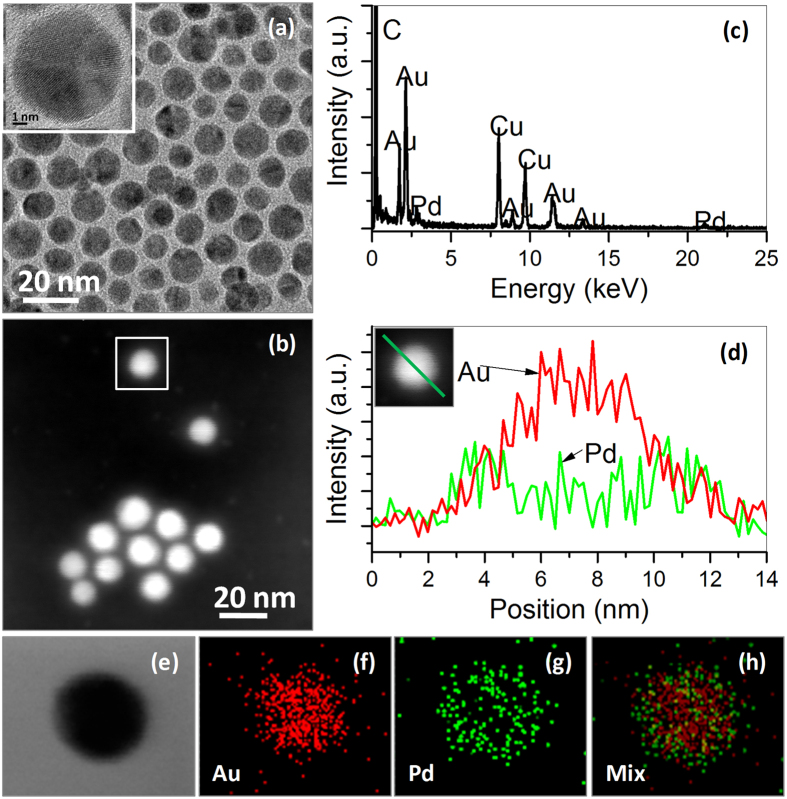
Core-shell Au-Pd nanoparticles. TEM image (**a**) STEM image (**b**) STEM-EDX analysis (**b**,**c**) elemental profiles in STEM mode (**d**) and nanoscale element mappings (**e**–**h**) of core-shell Au-Pd prepared in oleylamine at elevated temperature. Insert in (**a**) is the HRTEM image of a single Au-Pd particle.

Bimetallic core-shell Au-Pd nanoparticles were prepared using a seed-mediated growth strategy, which involves the synthesis of Au seed particles and the subsequent growth of a thin Pd layer in oleylamine^24^. Figure 1a, its insert, and 1b show the TEM, HRTEM, and STEM images of the as-prepared core-shell Au-Pd nanoparticles, respectively, which indicate that the core-shell particles are spherical with an average diameter of 12.2 nm. The EDX analysis in the STEM mode (Fig. 1c) of an arbitrary single particle boxed in Fig. 1b demonstrates that the particles as-prepared are composed of Au and Pd components, which have an atomic ratio of 6.4/1, well agreement with the Au/Pd molar ratio in their starting precursors. The formation of core-shell structure is confirmed by the elemental profiles of a single particle in the STEM mode. As shown in Fig. 1d, the signal of Au is confined to core region whereas the Pd signal is uniformly distributed throughout the entire particle. The nanoscale element mappings could also be used to infer the formation of core-shell Au-Pd structure. As revealed by Fig. 1e–h, the distributions of Au and Pd in the bimetallic nanoparticles are well in accordance to the element profiles, clearly supporting that the bimetallic Au-Pd nanoparticles thus obtained have core-shell constructions.

The XRD pattern of the as-prepared core-shell Au-Pd nanoparticles was exhibited in Figure S1 of Supplementary Information (SI), which manifests that the core-shell Au-Pd particles have a face-centered cubic (fcc) phase. In addition, their XRD pattern is more analogous to that of Au due to the dominant amount of Au in the core-shell products. The core-shell nanoparticles were subjected to XPS analyses for the determination of the chemical states of Au and Pd components. As shown in SI Figure S2a, a single doublet at 83.6 eV and 87.3 eV for Au in core-shell Au–Pd particles is the signature of zero valent Au^25^. The XPS 3d spectrum of Pd in core-shell products can be deconvoluted into two pairs of doublets (SI Figure S2b). The more intense doublet (at 335.1 eV and 340.5 eV) corresponds to Pd at zero valent state, while the second and weaker doublet, with binding energies higher than those of zero valent Pd could be assigned to Pd at oxidized state^25^,^26^.

**Figure 2:**
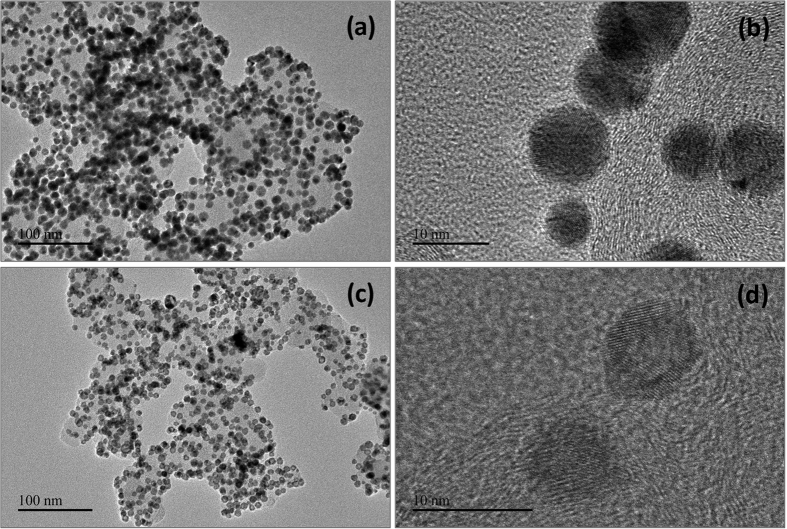
Core-shell Au-Pd and hollow Pt nanoparticles. TEM images (**a**,**c**) and HRTEM images (**b**,**d**) of bimetallic core-shell Au-Pd (**a**,**b**) and hollow structured Pt nanoparticles (**c**,**d**) supported on Vulcan carbon substrates.

The core-shell Au-Pd nanoparticles were supported on Vulcan carbon substrates and tested for catalytic activity for ORR at neutral electrolyte, e.g. phosphate buffer solution (PBS). As shown by Fig. 2a,b for the representative TEM and HRTEM images, the core-shell Au-Pd nanoparticles are well dispersed on the carbon substrates. The loading of the core-shell particles on carbon supports was fixed at 20 wt% of Pd. To benchmark the activity of core-shell Au-Pd nanoparticles as catalysts for ORR, Pt nanoparticles with hollow interiors supported on same Vulcan carbon substrates with mass ratio of 20% were also produced using the approaches we developed previously^27^. In brief, we firstly prepared core-shell Ag-Pt nanoparticles by successively reducing the AgNO_3_ and Pt(acac)_2_ in oleylamine at elevated temperature, and then loaded them on Vulcan carbon substrates, followed by agitating them with saturated NaCl solution to remove the Ag cores. The TEM and HRTEM images of the as-prepared carbon-supported hollow Pt nanostructures were presented in Fig. 2c,d, respectively, which show the brightness contrast between the core and shell regions and also suggest the well dispersion of hollow Pt nanoparticles with an average diameter of ca. 9.78 nm on the carbon substrates.

**Figure 3:**
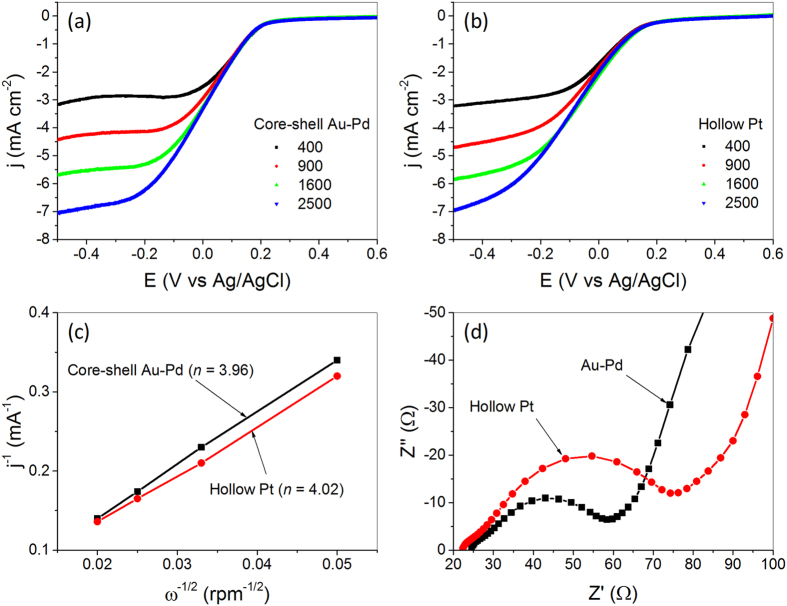
Electrochemical measurements. Rotating disk voltammograms for carbon-supported core-shell Au-Pd (**a**) and hollow structured Pt nanoparticles (**b**) measured in O_2_-saturated pH-neutral electrolyte; Koutechý-Levìch plots of carbon-supported core-shell Au-Pd and hollow Pt nanoparticles (**c**); Nyquist plots of electrochemistry impedance spectra for carbon-supported core-shell Au-Pd and hollow Pt nanoparticles measured in pH-neutral electrolyte.

The ORR kinetics and electron-transfer number for bimetallic core-shell Au-Pd and hollow structured Pt nanoparticles supported on carbon substrates were examined by rotating disk electrode (RDE) tests, which were performed in 50 mM O_2_-saturated PBS through changing the rotating speed (400–2500 rpm). It has been well known that the ORR activity of Pd metal is approximate one order of magnitude lower than that of Pt in acidic conditions^28^,^29^. However, as exhibited by Fig. 3a,b for the rotating disk voltammograms of core-shell Au-Pd and hollow structured Pt nanoparticles supported on carbon substrates, both the half-wave potentials and their corresponding kinetic current densities of bimetallic core-shell Au-Pd nanoparticles are quite close to those of hollow Pt nanostructures, indicating that they have comparable activity for ORR at neutral electrolyte. In addition, the half-wave potentials for core-shell Au-Pd nanoparticles are much higher than those recently reported for the cathode catalysts based on transition metal oxides. For example, this value is ca. 46 mV for core-shell Au-Pd nanoparticles at 1600 rpm, while only ca. −100 mV for Co_3_O_4_-doped activated carbon^21^, and ca. −160 mV for silver-tungsten carbide (Ag-WC) nanohybrids^30^, respectively. The Koutechý-Levìch plots for the carbon-supported core-shell Au-Pd and hollow structured Pt nanoparticles were given in Fig. 3c, which could be used to determine the number of electrons transferred in ORR. All of the Koutechý-Levìch plots yield straight lines, from which the electron-transfer number (*n*) for ORR on core-shell Au-Pd nanoparticles is calculated to be 3.96. This indicates that the ORR on core-shell Au-Pd nanoparticles in neutral electrolyte occurs by the same 4-electron reaction pathway as that on hollow Pt nanostructures (*n* = 4.02). Further, the electrochemical impedance spectroscopy (EIS) was adopted to explore the electrode reaction process for the core-shell Au-Pd and hollow Pt catalysts. As shown by Fig. 3d for the Nyquist plots, which were operated at an open circuit potential (OCP), in the high-frequency area, a depressed semicircle caused by the ionic migration, the charge transfer, and the resistance of the electrolyte can be observed. In the low-frequency area, the straight line with an angle of ca. 45° to the Z′-axis is found, which is the typical characteristic of the semi-finite diffusion (Warburg impedance) on the flat electrode. As exhibited by SI Figure S3, an equivalent circuit including three parts for porous electrode was employed to analyze the EIS result. The first part is the ohmic resistance (*R*_s_), which represents the electrolyte resistance between the cathode and the counter electrode surface, the second part is composed of an interface ohmic resistance (*R*_d_) and double layer capacitances (*CPE*_dl_) of the catalyst connected in parallel, and the third part consists of charge transfer resistance (*R*_ct_) connected in series with Warburg impedance (*W*) and pore adsorption capacitance (*CPE*_ad_) connected in parallel with them. SI Figure S4 and Table S1 show the fitted data of elements in the adopted equivalent circuit. The *R*_s_ of the hollow Pt catalyst is 20.97 Ω, which is lower than that of core-shell Au-Pd catalyst (24.11 Ω), this may be attributed to the higher conductivity of Pt materials. However, the *R*_d_ of the core-shell Au-Pd nanoparticles is 6.24 Ω, decreased by 78% compared to that of hollow Pt nanostructures (28.32 Ω). For the *R*_ct_, the characteristic for evaluating electrode reaction capability, in contrast to hollow Pt of 26.97 Ω, the core-shell Au-Pd catalysts decreased by 2.59 Ω. Further, the Warburg impedance of core-shell Au-Pd nanoparticles is much lower than that of hollow Pt nanostructures (19.44 Ω for core-shell Au-Pd and 69.75 Ω for hollow Pt, respectively). Obviously, the electrode with bimetallic core-shell Au-Pd nanoparticles displays much lower bulk resistance than that of the electrode with hollow Pt nanostructures.

As has been demonstrated previously, in core-shell Au-Pd nanoparticles, the Au cores impose strong lattice strain effect on the thin Pd shells due to the lattice mismatch. The lattice strain between the Au cores and Pd shells would affect the d-band center of the latter, and therefore alter their reactivity by changing the electronic properties of the Pd atoms^24^,^31^. In addition, the difference in electronegativities of the Au core (2.54) and the Pd (2.20) shell may imply potential electron-withdrawing effect from Au cores to the neighboring Pd shells, and this electronic coupling could also induce the change of the electronic structure of Pd atoms. In principle, an active ORR electrocatalyst should have the capability to balance both the bond-breaking and bond-making steps in a common ORR process^32^,^33^. For core-shell Au-Pd nanoparticles, although it is difficult to quantitatively differentiate the contribution of lattice strain and electronic coupling in catalyzing ORR, their appropriate d-band center or electronic structure may have collectively balanced both the two key steps occurred during the oxygen reduction, and thus offering the improved ORR catalytic activity compared with the hollow Pt nanostructures.

**Figure 4:**
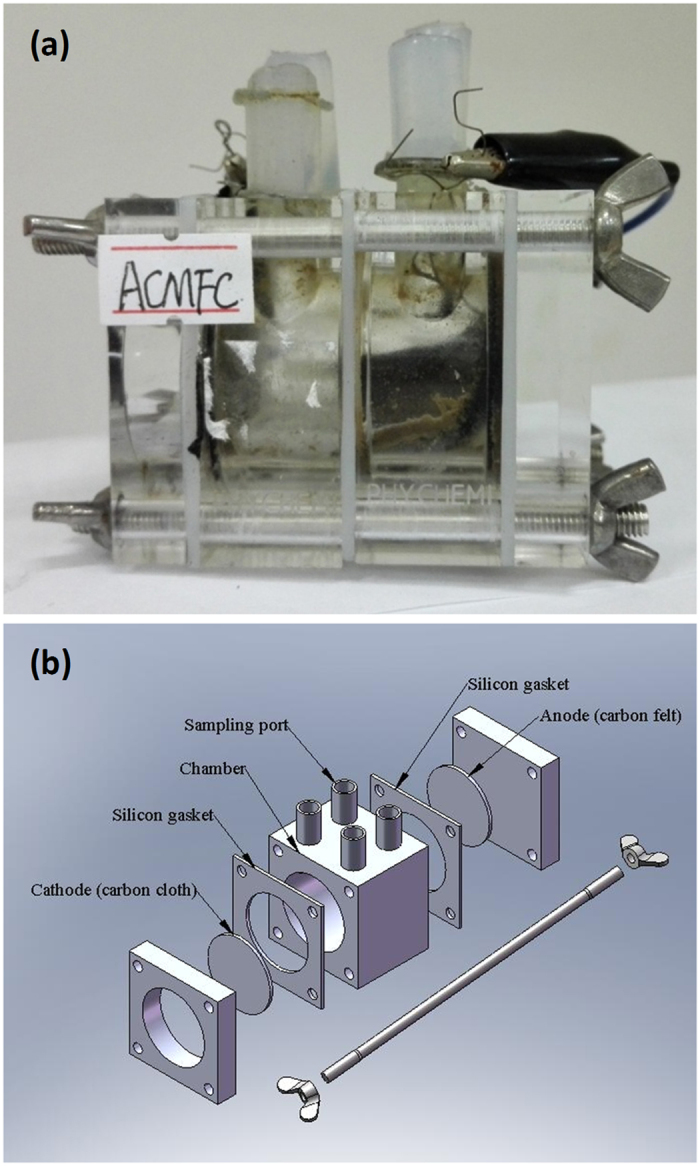
MFC assemblies. The practical construction (**a**) and schematic illustration (**b**) of a single-chambered microbial fuel cell.

**Figure 5:**
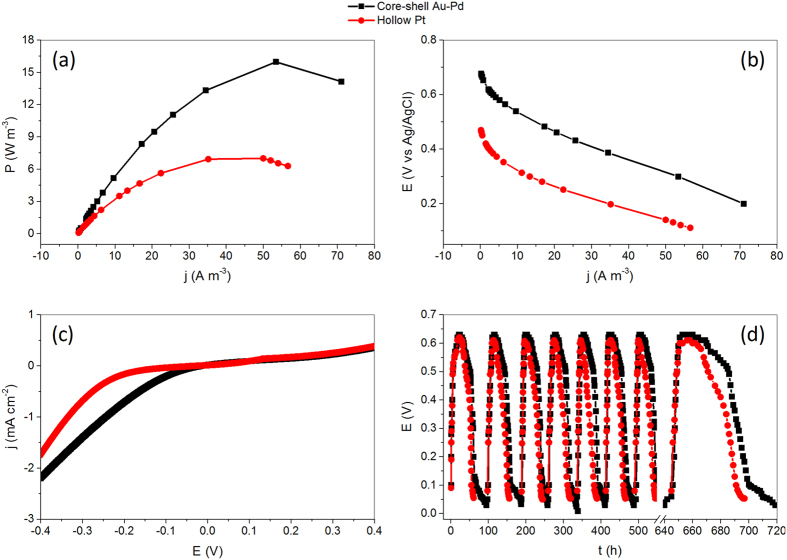
Catalyst Performance. A comparison of the power density (**a**) electrode polarization (**b**) LSVs (**c**) and voltage trends over time (**d**) for the core-shell Au-Pd and hollow structured Pt nanoparticles as cathode catalysts in a single-chambered MFC.

We assembled a single-chamber MFC to assess the performance of the core-shell cathode catalyst, which involves a carbon cloth at cathode, a carbon felt at anode, two silicon gaskets, one sample chamber, and four sampling ports, as displayed by Fig. 4a,b for its practical construction and schematic illustration, respectively. With electrochemically active bacteria fed periodically to the anode in 50 mM PBS electrolyte, the MFC using core-shell Au-Pd nanoparticles or hollow Pt nanostructures as air-cathode catalysts remains stable over 150 days. In the MFC studies, the core-shell Au-Pd cathode generates a maximum power density of ca. 16.0 W m^−3^, much higher than that of hollow structured Pt cathode (7.1 W m^−3^), as indicated by Fig. 5a. Given that the electrochemically active bacteria remain same at anode, the power output of the MFC should be governed by the cathodic ORR performance. The polarization curves of the core-shell Au-Pd and hollow structured Pt cathodes at different current densities were exhibited in Fig. 5b. The cathode voltages of core-shell Au-Pd nanoparticles are higher than those of hollow Pt nanostructures with the increase of current densities, showing that the core-shell Au-Pd nanoparticles have higher ORR catalytic performance than that of hollow Pt nanostructures. This result is in accord with the phenomenon observed from the power density curves, and clearly manifests that the core-shell Au-Pd nanoparticles could be used in MFCs as a good alternative to the Pt-based cathode catalysts for the production of electricity without losing power efficiency and stability. Figure 5c shows the results of the LSVs for the core-shell Au-Pd and hollow Pt cathode catalysts after running the MFCs for more than 150 days, which obviously indicate that the core-shell Au-Pd nanoparticles have higher current density than that of hollow Pt particles. At the potential of −0.2 V, the current density of core-shell Au-Pd reaches 0.77 mA cm^−2^, which is 78% higher than that of the hollow Pt, indicating that the core-shell Au-Pd particles is more beneficial for the ORR on the electrode. Further, The MFC with core-shell Au-Pd cathode catalysts demonstrates almost unchanged performance over the 8 cycles, while the MFC with the hollow Pt has a slightly decreasing voltage trend over time (Fig. 5d), again supporting the advantages of core-shell Au-Pd nanoparticles in long-term durability as cathode catalysts for MFCs.

In summary, we investigated the application of bimetallic core-shell Au-Pd nanoparticles as cathode catalysts in microbial fuel cells. Through the comparison with hollow structured Pt nanoparticles as cathode catalysts, we found that the bimetallic core-shell Au-Pd nanoparticles are more suitable for oxygen reduction reaction in a neutral condition. The maximum power density generated in a membraneless single-chamber microbial fuel cell running on wastewater with core-shell Au-Pd as cathode catalysts is ca. 16.0 W m^−3^ and remains stable over 150 days. Next, by optimizing both the size of Au cores and thickness of the Pd shells, further enhancement of the core-shell nanomaterials in MFCs might be expected.

## Methods

### General materials

Silver nitrate (AgNO_3_, ACS reagent, ≥99.0%), palladium(II) acetylacetonate (Pd(acac)_2_, 99%), platinum(II) acetylacetonate (Pt(acac)_2_, 97%), gold(III) chloride trihydrate (HAuCl_4_·3H_2_O, ACS reagent, ≥49.0% Au basis), oleylamine (70%, technical grade) and Nafion 117 solution (5% in a mixture of lower aliphatic alcohols and water) were purchased from Sigma-Aldrich. Ethanol (>99.7%), methanol (>99%), toluene (>99.5%) and perchloric acid solution (70%) were purchased from Beijing Chemical Works. Vulcan XC-72 carbon powders (XC-72C with BET surface area of 250 m^2^ g^−1^ and average particle size of ca. 40–50 nm) were purchased from Cabot. All chemicals were used as received.

### Bimetallic core-shell Au-Pd nanoparticles by the direct growth of a thin Pd shell on the Au seeds

Typically, 0.1 mmol of HAuCl_4_·3H_2_O was dissolved in 10 mL of oleylamine. The solution was heated to 150 °C and kept at this temperature for 1 h to reduce the Au^3+^ ions by oleylamine. Then 0.015 mmol of Pd(acac)_2_ was swiftly added to the colloidal solution of Au seeds at 150 °C, and the mixture was continuously kept at this temperature for 2 h, leading to the formation of core-shell Au-Pd nanoparticles. After the reaction, the core-shell Au-Pd nanoparticles were precipitated by methanol, followed by centrifugation and washing with methanol, then re-dispersed in 10 mL of toluene. Subsequently, a calculated amount of Vulcan carbon powders was added to the toluene solution of core-shell Au-Pd nanoparticles. After stirring the mixture for 24 h, the core-shell Au-Pd nanoparticles supported on carbin substrates (Pd mass ratio of 20%) were collected by centrifugation, which were then refluxed in acetic acid for 3 h at 120 °C to remove the oleylamine from the particle surface. After refluxing, the carbon-supported core-shell Au-Pd nanoparticles collected by centrifugation, washed thrice with water, and then dried at room temperature in vacuum.

### Hollow Pt nanostructures supported on carbon substrates

For the synthesis of carbon-supported hollow Pt nanostructures, core-shell Ag-Pt nanoparticles were firstly prepared. In detail, 68 mg of AgNO_3_ was added to 20 mL of oleylamine, and then the mixture was heated to 165 °C and kept this temperature under flowing N_2_ for 1 h for to reduce the Ag(I) ions. Then 79 mg of Pt(acac)_2_ was added swiftly, followed by heating at 165 °C for 2 h. After reaction, the core-shell Ag-Pt nanoparticles were precipitated with methanol, centrifugation, washing with methanol, and re-dispersed in 20 mL of toluene. Afterwards, the core-shell Ag-Pt nanoparticles were loaded on the carbon substrates (20% mass ratio of Pt) using the strategy developed for the preparation of carbon-supported core-shell Au-Pd nanoparticles. Finally, to remove the Ag cores from the core-shell Ag-Pt nanoparticles, 20 mg of carbon-supported core-shell Ag-Pt samples was added into 20 mL of saturated NaCl solution. The mixture was aged for 24 h under vigorous stirring at room temperature. Then the resulting carbon-supported hollow Pt nanostructures were collected by centrifugation, washed thrice with water, and then dried at room temperature in vacuum.

### Characterizations and electrochemical measurements of the nanoparticles

Transmission electron microscopy (TEM), high-resolution TEM (HRTEM), and scanning transmission electron microscopy (STEM) were performed on a JEOL JEM-2010F electron microscope operated at 200 kV. An energy dispersive X-ray spectroscopy (EDX) analyzer attached to the TEM operating in STEM mode was used to determine the chemical compositions as well as their distribution in the nanoparticles. For the TEM measurements, a drop of the nanoparticle solution was dispensed onto a 3-mm carbon-coated copper grid, and excessive solution was removed by an absorbent paper. Then the sample was dried under vacuum at room temperature. Powder X-ray diffraction (XRD) patterns were recorded on a Bruker D8 diffractometer using Cu K_α_ radiation (λ = 0.154056 nm). X-ray photoelectron spectra (XPS) were collected using a Thermo Scientific K-Alpha XPS spectrometer.

Electrochemical measurements were carried out in a standard three-electrode cell, which was connected to a Bio-logic VMP3 (with EC-lab software version 9.56) potentiostat. A leak-free Ag/AgCl (saturated with KCl) electrode was used as the reference. The counter electrode is a platinum mesh (1 × 1 cm^2^) attached to a platinum wire.

The working electrode was a thin layer of Nafion-impregnated catalyst cast on a vitreous carbon disk. This electrode was prepared by ultrasonically dispersing 5 mg of the carbon-supported nanoparticles in 1 mL of ethanol containing 0.05 mL of Nafion solution. A calculated volume of the ink was dispensed onto the 5 mm glassy carbon disk electrode to produce a nominal catalyst loading of 51 μg cm^−2^ (Pd basis). The carbon electrode was then dried in a stream of warm air at 70 °C for 1 h.

The performance of carbon-supported core-shell Au-Pd and hollow Pt nanoparticles in room temperature ORR was evaluated in 50 mM phosphate buffer solution (PBS) using a glass carbon rotating disk electrode (RDE) at different rotating rates (400, 900, 1600, and 2500 rpm). The linear sweep voltammetry (LSV) was tested from 0.4 V to −0.4 V with a scan rate of 10 mV s^−1^ in the 50 mM PBS. Negative-going LSVs were recorded from −0.5 to 0.6 V at a rate of 10 mV s^−1^ in the presence of bubbling ultra-pure oxygen to maintain a saturated oxygen atmosphere near the working electrode. In the ORR polarization curve, for each catalyst (carbon-supported core-shell Au-Pd and hollow Pt nanoparticles), the measured current density was normalized in reference to the surface area of the electrode to obtain the specific activities.

### Microbial fuel cell (MFC) performance tests

The single-chamber MFCs used in this work were constructed with a cylindrical chamber (28 mL) and an electrode spacing of 4 cm as previously described^34^. The anode electrodes with a project area of 9 cm^2^ were made of PAN-Carbon and graphite felt, while, according to the literature^35^, the cathode electrodes with different as-prepared catalysts (0.5 mg cm^−2^) were made of carbon cloths (Hesen electric co., ltd, Shanghai, China) with a 7 cm^2^ projected surface area. Titanium wire was used as cathode and anode leads. The acrylic plates, electrodes, and membrane were assembled with a silicon gasket to prevent leakage. A 1000 Ω resistor (except as noted) was routinely used as the load resistor.

The enrichment and adaptation of the electrochemically active bacteria in the air-cathode MFCs were performed in batch mode, and carried out for over 150 days. Activated sludge was obtained from the Jingye River, South China Agricultural University, Guangzhou, China. A medium consisting of a 50 mM PBS, vitamins, nutrients, minerals and 1 g L^−1^ of sodium acetate was used as the substrate to feed the reactors. As the voltage decreased to less than 30 mV, the reactor was refilled each time to form a complete of operation cycle.

The external circuit voltage (E) was measured by data acquisition card (model, ZP1001) from Guangzhou NXP Ltd. Power density curves and polarisation curves were obtained by varying the external resistance from 90000 to 50 Ω with a time interval of 10 min using resistor substitution boxes (The Tianshui Great Wall Electrical Instrument Co., Ltd. China). Current density (*I*), power density (*P*), the battery electromotive force (*U*) and battery internal resistance (*r*) were calculated by the following formulas:


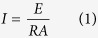



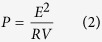



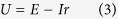


where, *R* is external resistance; *A* is the apparent area of the cathode material; *V* is volume of the anode chamber.

Electrochemical impedance spectroscopy (EIS) of cathodes was carried out over a frequency range of 200 kHz–10 mHz at open circuit potential with a sinusoidal perturbation signal amplitude of 10 mV using a potentiostat (Autolab PGSTAT 302N, Metrohm, Switzerland). Equivalent circuits were used to analyze the nyquist plots by NOVA 1.7.

## Additional Information

**How to cite this article**: Yang, G. *et al.* Core-shell Au-Pd nanoparticles as cathode catalysts for microbial fuel cell applications. *Sci. Rep.* **6**, 35252; doi: 10.1038/srep35252 (2016).

## Supplementary Material

Supplementary Information

## References

[b1] Rinaldi A. *et al.* Engineering materials and biology to boost performance of microbial fuel cells: a critical review. Energy Environ. Sci. 1, 417–429 (2008).

[b2] Franks A. E. & Nevin K. P. Microbial fuel cells: a current review. Energies 3, 899–919 (2010).

[b3] Dewan A., Donovan C., Heo D. & Beyenal H. Evaluating the performance of microbial fuel cells powering electronic devices. J. Power Sources 195, 90–96 (2010).

[b4] Logan B. E. & Elimelech M. Membrane-based processes for sustainable power generation using water and wastewater. Nature 488, 313–319 (2012). 22895336 10.1038/nature11477

[b5] Thomas Y. R. J. *et al.* A single sediment-microbial fuel cell powering a wireless telecommunication system. J. Power Sources 241, 703–708 (2013).

[b6] Ledezma P., Stinchcombe A., Greenman J. & Ieropoulos I. The first self-sustainable microbial fuel cell stack. Phys. Chem. Chem. Phys. 15, 2278–2281 (2013). 23321677 10.1039/c2cp44548d

[b7] Papaharalabos G. *et al.* Increased power output from micro porous layer (MPL) cathode microbial fuel cells (MFC). Int. J. Hydrogen Energy 38, 11552–11558 (2013).

[b8] Ieropoulos I. A. *et al.* Waste to real energy: The first MFC powered mobile phone. Phys. Chem. Chem. Phys. 15, 15312–15316 (2013). 23939246 10.1039/c3cp52889h

[b9] Tommasi T., Chiolerio A., Crepaldi M. & Demarchi D. A microbial fuel cell powering an all-digital piezoresistive wireless sensor system. Microsyst. Technol. 20, 1023–1033 (2014).

[b10] Wang H., Park J.-D. & Ren Z. J. Practical energy harvesting for microbial fuel cells: a review. Environ. Sci. Technol. 49, 3267–3277 (2015). 25670167 10.1021/es5047765

[b11] Logan B. E. *et al.* Microbial fuel cells: methodology and technology. Environ. Sci. technol. 40, 5181–5192 (2006). 16999087 10.1021/es0605016

[b12] Du Z., Li H. & Gu T. A state of the art review on microbial fuel cells: a promising technology for wastewater treatment and bioenergy. Biotechnol. Adv. 25, 464–282 (2007). 17582720 10.1016/j.biotechadv.2007.05.004

[b13] Feng Y. *et al.* Effects of sulfide on microbial fuel cells with platinum and nitrogen-doped carbon powder cathodes. Biosens. Bioelectron. 35, 413–415 (2012). 22424752 10.1016/j.bios.2011.08.030

[b14] Erable B., Féron D. & Bergel A. Microbial catalysis of the oxygen reduction reaction for microbial fuel cells: a review. ChemSusChem 5, 975–987 (2012). 22615123 10.1002/cssc.201100836

[b15] Santoro C. *et al.* Activated carbon nanofibers (ACNF) as cathode for single chamber microbial fuel cells (SCMFCs). J. Power Sources 243, 499–507 (2013).

[b16] Harnisch F. & Schröder U. From MFC to MXC: chemical and biological cathodes and their potential for microbial bioelectrochemical systems. Chem. Soc. Rev. 39, 4433–4448 (2010). 20830322 10.1039/c003068f

[b17] Liew K. B. *et al.* Non-Pt catalyst as oxygen reduction reaction in microbial fuel cells: a review. Int. J. Hydrogen Energy 39, 4870–4883 (2014).

[b18] Dong H., Yu H., Wang X., Zhou Q. & Feng J. A novel structure of scalable air-cathode without Nafion and Pt by rolling activated carbon and PTFE as catalyst layer in microbial fuel cell. Water Res. 46, 5777–5787 (2012). 22939222 10.1016/j.watres.2012.08.005

[b19] Cheng S. & Wu J. Air-cathode preparation with activated carbon as catalyst, PTFE as binder and nickel foam as current collector for microbial fuel cells. Bioelectrochemistry 92, 22–26 (2013). 23567144 10.1016/j.bioelechem.2013.03.001

[b20] Wang X. *et al.* Acidic and alkaline pretreatments of activated carbon and their effects on the performance of air-cathodes in microbial fuel cells. Bioresource Technol. 144, 632–636 (2013). 10.1016/j.biortech.2013.07.022 23890977

[b21] Ge B., Li K., Fu Z., Pu L. & Zhang X. The addition of ortho-hexagon nano spinel Co_3_O_4_ to improve the performance of activated carbon air cathode microbial fuel cell. Bioresource Technol. 195, 180–187 (2015). 10.1016/j.biortech.2015.06.054 26112347

[b22] Santoro C. *et al.* High catalytic activity and pollutants resistivity using Fe-AAPyr cathode catalyst for microbial fuel cell application. Sci. Rep. 5, 16596 (2015). 26563922 10.1038/srep16596 PMC4643260

[b23] Burkitt R., Whiffen T. R. & Yu E. H. Iron phthalocyanine and MnOx composite catalysts for microbial fuel cell applications. Appl. Catal. B: Environ. 181, 279–288 (2016).

[b24] Chen D., Li C., Liu H., Ye F. & Yang J. Core-shell Au@Pd nanoparticles with enhanced catalytic activity for oxygen reduction reaction *via* core-shell Au@Ag/Pd constructions. Sci. Rep. 5, 11949 (2015). 26144550 10.1038/srep11949 PMC4491719

[b25] Wagner C. D. *et al.* [*NIST Standard Reference Database 20*], (Version 3.2, Web Version).

[b26] Yang J., Lee J. Y. & Too H.-P. A general phase transfer protocol for synthesizing alkylamine-stabilized nanoparticles of noble metals. Anal. Chim. Acta 588, 34–41 (2007). 17386791 10.1016/j.aca.2007.01.061

[b27] Liu H., Ye F. & Yang J. A universal and cost-effective approach to the synthesis of carbon-supported noble metal nanoparticles with hollow interiors. Ind. Eng. Chem. Res. 53, 5925–5931 (2014).

[b28] Savadogo O. *et al.* New palladium alloys catalyst for the oxygen reduction reaction in an acid medium. Electrochem. Commun. 6, 105–109 (2004).

[b29] Mathiyarasu J. & Phani K. L. N. Carbon-supported palladium-cobalt-noble metal (Au, Ag, Pt) nanocatalysts as methanol tolerant oxygen-reduction cathode materials in DMFCs. J. Electrochem. Soc. 154, B1100–B1105 (2007).

[b30] Gong X.-B. *et al.* Silver-tungsten carbide nanohybrid for efficient electrocatalysis of oxygen reduction reaction in microbial fuel cell. J. Power Sources 225, 330–337 (2013).

[b31] Chen D., Li J., Cui P., Liu H. & Yang J. Gold-catalyzed formation of core–shell gold–palladium nanoparticles with palladium shells up to three atomic layers. J. Mater. Chem. A 4, 3813–3821 (2016).

[b32] Stamenković V. R. *et al.* Improved oxygen reduction activity on Pt_3_Ni(111) via increased surface site availability. Science 315, 493–497 (2007). 17218494 10.1126/science.1135941

[b33] Stamenković V. R. *et al.* Trends in electrocatalysis on extended and nanoscale Pt-bimetallic alloy surfaces. Nat. Mater. 6, 241–247 (2007). 17310139 10.1038/nmat1840

[b34] Liu H. & Logan B. E. Electricity generation using an air-cathode single chamber microbial fuel cell in the presence and absence of a proton exchange membrane. Environ. Sci. Technol. 38, 4040–4046 (2004). 15298217 10.1021/es0499344

[b35] Cheng S., Liu H. & Logan B. E. Increased performance of single-chamber microbial fuel cells using an improved cathode structure. Electrochem. Commun. 8, 489–494 (2006).

