# Supplementary material for: Core-shell Au-Pd nanoparticles as cathode catalysts for microbial fuel cell applications
Source: Sci Rep. 2016 Oct 13;6:35252. doi: 10.1038/srep35252 (PMC5062343; doi:10.1038/srep35252)
Supplement: Supplementary Information [file srep35252-s1.doc]

Supplementary Information

**Core-shell Au-Pd nanoparticles as cathode catalysts for microbial fuel cell applications**

Gaixiu Yang^1,2^, Dong Chen^3^, Pengmei Lv^1,2^, Xiaoying Kong^1,2^, Yongming Sun^1,2^,*, Zhongming Wang^1,2^, Zhenhong Yuan^1,2^, Hui Liu^3^ & Jun Yang^3^,*

^1^Key Laboratory of Renewable Energy, Chinese Academy of Sciences, Guangzhou Institute of Energy Conversion, Chinese Academy of Sciences, Guangzhou 510640, China. Fax: 86-20-3702 9689; Tel: 86-20- 8705 7009; E-mail: sunym@ms.giec.ac.cn

^2^Guangdong Key Laboratory of New and Renewable Energy Research and Development, Guangzhou Institute of Energy Conversion, Chinese Academy of Sciences, Guangzhou 510640, China

^3^State Key Laboratory of Multiphase Complex Systems, Institute of Process Engineering, Chinese Academy of Sciences, Beijing 100190, China. Fax: 86-10-8254 4814; Tel: 86-10-8254 4915; E-mail: [jyang@ipe.ac.cn](mailto:jyang@mail.ipe.ac.cn)

Financial support from the Science and Technology Service Network Initiative (No.: KFJ-Ew-STS-138), Science and Technology Planing Project of Guangdong Province (No.: 2015B020241002), National Natural Science Foundation of China (Nos.: 21376247, 21506225, 21573240) is gratefully acknowledged.

**Figure S1  Crystal structure of core-shell Au-Pd products.** X-ray diffraction (XRD) pattern of core-shell Au-Pd nanoparticles. The references for face centered cubic Au and Pd crystals (JCPDS Card File 040784 and 870643, respectively) are also displayed.

**Figure S2  XPS spectra.** The 4f XPS spectrum of Au (a) and 3d XPS spectrum of Pd (b) in core-shell Au-Pd nanoparticles as-prepared in oleylamine at elevated temperature.


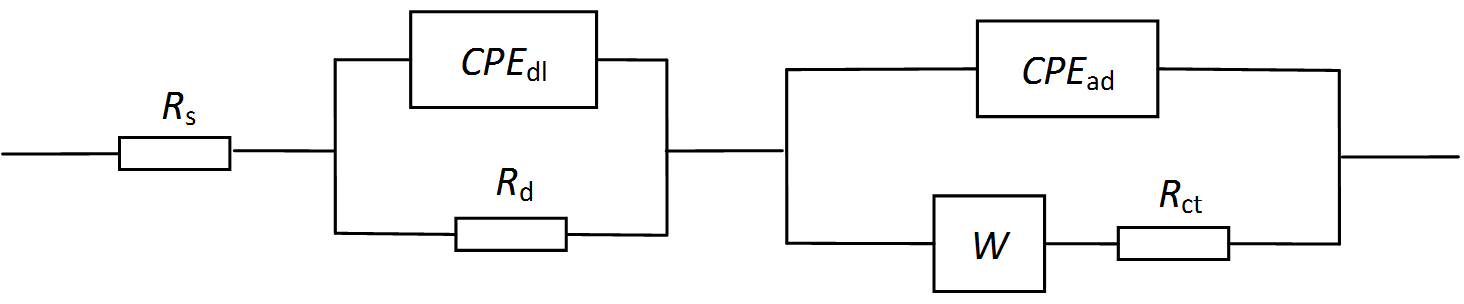


**Figure S3  Equivalent circuit.** The equivalent circuit for the electrochemical system where the symbols represent the elements of ohmic resistance (*R*_s_), interface ohmic resistance (*R*_d_), double layer capacitances (*CPE*_dl_), pore adsorption capacitance (*CPE*_ad_), Warburg impedance (*W*), and charge-transfer resistance (*R*_ct_), respectively.

**Figure S4  Fitting data.** The fitting data from the equivalent circuit for the Nyquist plots of EIS by core-shell Au-Pd nanoparticles and hollow Pt nanostructures.

**Table S1. Fitting results of different cathodes based on the equivalent circuit in Figure S1.**

| **Element** | **Au-Pd** | | **Hollow Pt** |
| --- | --- | --- | --- |
| *R*_s_ (Ω) | 24.11 | 20.97 | |
| *R*_d_ (Ω) | 6.24 | 28.32 | |
| *W* (Ω) | 19.44 | 69.75 | |
| *CPE*_dl_-T | 6.57×10^-5^ | 4.21×10^-5^ | |
| *CPE*_dl_-P | 0.68 | 0.98 | |
| *CPE*_ad_-T | 5.10×10^-5^ | 0.0018 | |
| *CPE*_ad_-P | 0.84 | 0.43 | |
| *R*_ct_ (Ω) | 24.38 | 26.97 | |
